# LidPose: Real-Time 3D Human Pose Estimation in Sparse Lidar Point Clouds with Non-Repetitive Circular Scanning Pattern

**DOI:** 10.3390/s24113427

**Published:** 2024-05-26

**Authors:** Lóránt Kovács, Balázs M. Bódis, Csaba Benedek

**Affiliations:** 1 HUN-REN Institute for Computer Science and Control (SZTAKI), Kende utca 13-17, H-1111 Budapest, Hungary; 2 Faculty of Information Technology and Bionics, Pázmány Péter Catholic University, Práter utca 50/A, H-1083 Budapest, Hungary

**Keywords:** lidar-only 3D human pose estimation, real-time surveillance, point cloud, rosetta pattern non-repetitive circular scanning, NRCS lidar

## Abstract

In this paper, we propose a novel, vision-transformer-based end-to-end pose estimation method, LidPose, for real-time human skeleton estimation in non-repetitive circular scanning (NRCS) lidar point clouds. Building on the ViTPose architecture, we introduce novel adaptations to address the unique properties of NRCS lidars, namely, the sparsity and unusual rosetta-like scanning pattern. The proposed method addresses a common issue of NRCS lidar-based perception, namely, the sparsity of the measurement, which needs balancing between the spatial and temporal resolution of the recorded data for efficient analysis of various phenomena. LidPose utilizes foreground and background segmentation techniques for the NRCS lidar sensor to select a region of interest (RoI), making LidPose a complete end-to-end approach to moving pedestrian detection and skeleton fitting from raw NRCS lidar measurement sequences captured by a static sensor for surveillance scenarios. To evaluate the method, we have created a novel, real-world, multi-modal dataset, containing camera images and lidar point clouds from a Livox Avia sensor, with annotated 2D and 3D human skeleton ground truth.

## 1. Introduction

Human pose estimation is an essential task in machine perception and has several real-world applications, among others in robotics [1], security and surveillance [2,3], autonomous driving [4], human–computer interaction [5], sports performance analysis [6], healthcare [7], forensic science [8], and entertainment and gaming [9].

The main task of pose estimation is to localize the anatomical keypoints of the human body in three-dimensional space.

In this paper, we demonstrate the efficiency of using a cost-efficient lidar sensor, called Livox Avia [10], for human pose estimation. The addressed lidar sensor has a unique *non-repetitive circular scanning (NRCS)* pattern. We propose a vision-transformer-based, neural-network-based approach to detect and fit human skeleton models solely based on the NRCS lidar data.

### 1.1. Related Works

Human pose estimation is usually solved by camera-based methods [11,12,13] in the image space. However, such solutions are inherently limited by the camera’s incapability to directly measure distance, the high sensitivity of the captured images to various lighting and weather conditions, and the varying visual appearances of real-world objects. The consideration of additional depth information can increase the pose estimation robustness, as shown in [1], which uses an RGBD camera for 3D human pose estimation, outperforming camera-based 3D estimators and depth-only methods. In applications, where privacy is a serious concern, lidar-based human surveillance can be efficiently applied, as the observed people cannot be identified by an observer in the sparse point cloud.

For 3D human pose estimation, Refs. [4,14] use semi-supervised learning approaches, where the 2D annotations are lifted to the 3D space and the methods use the fusion of camera images and lidar point clouds.

Aside from camera-based methods, the human pose estimation task has also been addressed by processing lidar measurements.

Lidar-based human pose estimation faces several challenges, including sparse data representation and limited spatial resolution. These originate from lidar’s working method, as the sensors have a limited field of view (FoV), being either only in the vertical axis or both in the vertical and horizontal directions, leading to incomplete and/or sparse point clouds. Upon proposing a lidar-based solution these issues have to be addressed.

In [15], the authors proposed a method for 3D human pose and shape estimation from a point cloud sequence. Although that method can regress the 3D mesh of a human body, it does not make predictions about the underlying human skeleton. Similarly, LiveHPS proposed in [16] also estimates the human pose and shape using a point cloud sequence, recorded with a rotating multi-beam (RMB) lidar. Although this method extracts point-wise features and predicts the human body joint positions, it uses the inertial measurement unit (IMU) sensor’s data alongside the lidar point clouds for the pose detection, similarly to the LIP method described in [17]. Dense depth images can be used to estimate human pose, as shown in [18], using a deep graph convolutional neural network-based network [19]. The input of this method is a point cloud, derived from the 2D depth images recorded with a depth camera. The method relies on the denseness of the point cloud, which does not make it suitable to process sparse point clouds recorded with an NRCS lidar sensor.

The *LPFormer* method [20] works on point clouds recorded with RMB lidars, and it is developed and tested on the Waymo Open Dataset [21]. However, the technique exploits particular measurement modalities apart from the 3D point coordinates, namely, the intensity, elongation, and the timestamp associated with each lidar point; such requirements are a limitation for using the LPFormer method with different lidar types, including the NRCS lidar sensors.

Vision transformers have made significant progress and successes recently in several computer vision tasks [22,23], such as object detection [24], image generation [25,26,27], but also in pose estimation [20,28,29]. A notable approach for camera-based human pose estimation is ViTPose [28], a vision-transformer-based human pose estimator. The method yields state-of-the-art results while running in real time on camera images. Given the attractive properties of ViTPose [28] and the fact that transformers [22] handle sparse data better than the mostly convolution-based skeleton estimation methods [30,31,32], we propose here a modified ViTPose architecture to process the sparse lidar input data for 3D human pose estimation, expecting that the transformer-based [22] approach is capable of handling the sparse lidar input data more efficiently than the mostly convolution-based skeleton detection methods [30,31,32].

### 1.2. NRCS Lidar Sensor

The Livox Avia [10] sensor has six lidar beams organized in a linear beam array, which is moved and rotated inside the sensor to scan its FoV (horizontal: 70°, vertical: 77°, with 0.05° angular precision). The sensor has a detection range of up to 320 m if the target object reflects at least 80% of the light and 190 m at 10% object reflectivity. The sensor’s distance error (1σ) is less than 2 cm at 20 m. The angular error (1σ) is smaller than 0.05° [33].

Unlike most rotating and scanning lidars (e.g., Velodyne HDL-64, Ouster OS sensor family), which boost a repetitive scanning pattern, the Avia does not repeat the exact scanning paths in every frame, but instead, the lasers cover new parts of the field of view. This key difference is both beneficial and has some disadvantages. NRCS lidars cover the complete FoV over time, providing rich spatial information, especially in static scenarios. On the other hand, because the same region is scanned less frequently than by using “regular” RMB lidars, dynamic objects, such as humans, may cause challenges as they induce heavy motion blur in the recorded NRCS point clouds.

The used Livox Avia is an active laser-based sensor, working efficiently under different lightning and illumination conditions. However, this robustness decreases in harsh weather conditions: the sensor has weaker performance in fog, snow, or heavy rain [34] In dense fog or heavy rain, the water droplets reflect the emitted laser beams by creating false distance measurements from the observed scene. A possible approach for weather-related point cloud denoising is the WeatherNet network, described in [35].

As demonstrated in [36,37], the NRCS approach is suitable for a wide range of perception use cases from *simultaneous localization and mapping (SLAM)* to low-speed autonomous driving. The NRCS lidar sensor offers real-time scene analysis capabilities, and it is available on the market at cheaper prices compared to other lidar sensors [38]. The sensor continuously records distance measurements with accompanying timestamps as it follows a non-repetitive circular pattern within its FoV. In this paper, the Livox Avia sensor gathers approximately 240,000 points per second [33].

**Figure 1:**
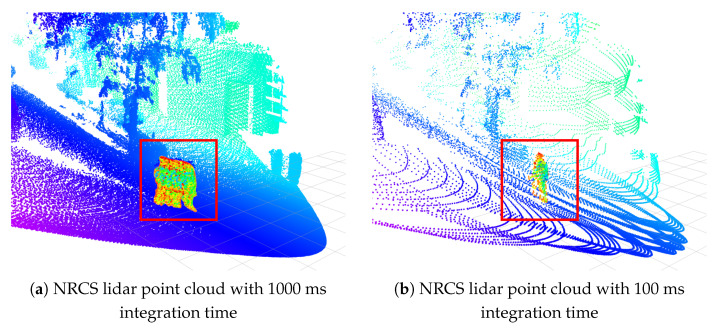
Point cloud sample recorded on the same scene with different integration times using the NRCS lidar. The sparse point cloud can be seen on the left, while a denser cloud is visible on the right. Note that while increased integration time brings more density, it also introduces motion blur on dynamic objects, as shown with the moving pedestrian marked with the red rectangle. The pedestrian’s points are colored with the lidar intensity, the background is colored by the y-axis value.

By establishing a consistent integration time, the points collected sequentially can be grouped into distinct lidar time frames. A primary challenge lies in effectively managing the spatial and temporal resolution of the captured point cloud data. While extending the integration time permits a broader coverage of the FoV by the laser beams, resulting in an increased spatial resolution of the measurement frame, the movements of dynamic objects within the observed area introduce diverse motion artifacts (e.g., blurred pedestrian silhouettes, as shown in Figure 1a), thereby complicating their precise perception.

Conversely, when measurements are gathered within a shorter time window (e.g., 100 ms), the resultant point clouds exhibit sparsity, resulting in reduced spatial details across the FoV: an example frame containing ∼24,000 points collected within 100 ms is depicted in Figure 1b.

Another sensor-specific property of the recorded data is the inhomogeneous point cloud density.

**Figure 2:**
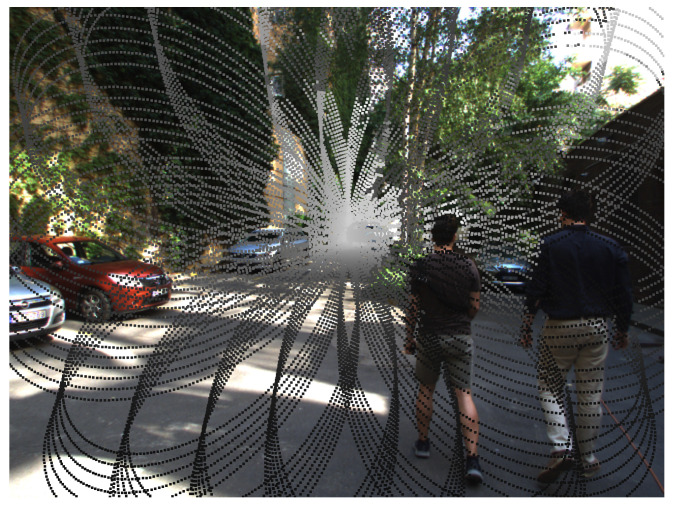
Non-repetitive circular scanning (NRCS) lidar point cloud with 100 ms integration time represented as a 2D range image overlaid on a sample camera image. NRCS lidar point cloud is colored by the distance: the lighter the point’s color, the greater its distance.

More specifically, while the center of the field of view is scanned in every rotation of the pattern, outer regions are sampled less frequently, as demonstrated in Figure 2. This particular, inhomogeneous point density distribution makes it difficult to apply existing lidar point cloud processing approaches on NRCS lidar measurement sequences [39]. Note that apart from depth data, the sensor also records the reflection intensity of the laser beams in the range 0–100% according to the Lambertian reflection model [33].

### 1.3. Contributions and Paper Outline

Our contributions are the following:

- We propose a novel, real-time, end-to-end 3D human pose estimation method using only sparse NRCS lidar point clouds.
- A new dataset is created including synchronized and calibrated lidar and camera data along with human pose annotations. Note that in our lidar-only approach, camera images are only used for parameter training and validation of the results.
- Using this dataset, we demonstrate in multiple experiments the proper input data and network architecture to achieve accurate and real-time 3D human pose estimation in NRCS lidar point clouds.

The outline of the paper is as follows. In Section 2, the proposed method is introduced in detail, including its processing steps, various input types, and its 2D and 3D prediction outputs. Section 3 describes our new dataset created for the training and testing of the proposed methods. In Section 4, we describe and analyze the quantitative and qualitative evaluation results. Section 5 concludes the paper and provides future work directions.

## 2. Proposed Method

The goal of the proposed method is to detect human poses in lidar frames recorded by an NRCS lidar sensor. The proposed method is an end-to-end solution for moving person detection and pose estimation in a surveillance use case, where the NRCS lidar sensor is placed in a fixed position. The human pose is represented by an ordered list of anatomical keypoints, referred to as *joints* hereinafter.

The sensor’s non-repetitive circular scanning pattern presents a significant challenge: the scanning laser beams are unable to densely cover the entire FoV of the sensor within a data collection window. This limitation leads to numerous sparse and even empty regions within the individual lidar frames, particularly near the edges of the sensor’s FoV. Additionally, there is a noticeable inhomogeneous point density, as illustrated in Figure 1.

The human pose estimation task can be applied in surveillance applications, which demand real-time solutions. To address this need, our approach involves transforming the representation of the NRCS lidar point cloud from 3D Cartesian coordinates to a spherical polar coordinate system, similar to our previous works in [39,40]. We generate a 2D pixel grid by discretizing the horizontal and vertical FoVs, where each 3D point’s distance from the sensor is mapped to a pixel determined by corresponding azimuth and elevation values. The polar direction and azimuth angles correspond to the horizontal and vertical pixel coordinates, while the distance is encoded as the ‘gray’ value of the respective pixel. This process allows the subsequent steps of our proposed lidar-only 3D human pose estimation method to be developed within the domain of 2D range images.

Depending on the timing window of data collection, as illustrated in Figure 1, the range image of a specific lidar frame may contain numerous pixels with undefined range values due to the NRCS scanning pattern. The number of these undefined pixels depends on both the measurement integration time and the predefined dimensions of the range image. In our experiments, we leveraged the precision parameters of the Livox Avia sensor, mapping its FoV onto a 600×660 pixel grid, resulting in a spatial resolution of 8.5 pixels per degree. It is important to note that the density of the recorded valid-range values decreases towards the periphery of the range image due to the scanning technique: the scanning pattern crosses the sensor’s optical center more frequently than it covers the perimeter regions of the FoV. This 2D range image-based data representation facilitated the efficient and robust utilization of sparse lidar data.

The proposed method is based on the state-of-the-art ViTPose [28] human pose estimation method, working on camera images, based on a vision transformer (ViT) architecture [23], which was trained on the COCO dataset [41].

### 2.1. ViTPose

ViTPose is a deep-learning-based method for human skeleton estimation that can achieve real-time performance and outstanding estimation accuracy [28]. ViTPose works on images containing a single person with a tight crop. It has three main parts: network backbone, network head, and joint position reconstruction. The network’s backbone is a plain and non-hierarchical vision transformer. Its input is a camera image, cropped around the human subject. The backbone embeds the input data into tokens using patch embedding and down-sampling. These embedded tokens are fed to several transformer layers. Each of these layers consists of a *multi-head self-attention (MHSA)* layer and a *feed-forward network (FFN)*. The output of the transformer layer is processed by a decoder. ViTPose’s head is the decoder network, which processes the transformer blocks’ output in the feature space. It employs direct up-sampling with bilinear interpolation, which is followed by a *rectified linear unit (ReLU)* and a 3×3 convolution. The output of the network head is a set of heatmaps, one heatmap for each joint in a down-scaled and uniformed feature space. The heatmap encodes the likelihood of the presence of a joint at each pixel position. Thus, the local maxima of the heatmaps correspond to the possible joint locations. The third part of the method retrieves the final keypoint predictions from the heatmaps predicted by the network head and transforms the keypoint locations back to the original input image domain.

### 2.2. LidPose

**Figure 3:**
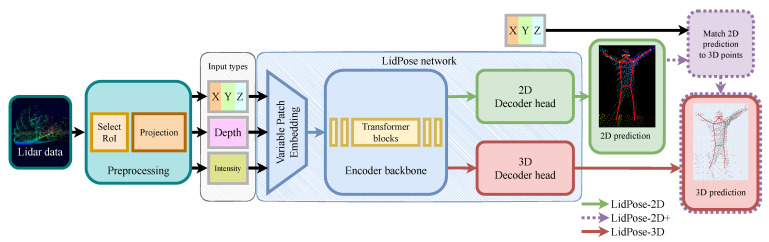
*LidPose* end-to-end solution. Lidar data: full lidar point cloud. Select RoI: selects the 3D points in the vicinity of the observed human. Projection stores the 3D point cloud in a 2D array. Input types: 3D XYZ coordinates (XYZ), depth (D), and intensity (I). LidPose network: both *LidPose–2D* and *LidPose–3D* use our patch-embedding module and the encoder backbone visible in blue. *LidPose–2D* and *LidPose–3D* use the corresponding decoder head and *LidPose–2D+* is calculated from the 2D prediction and the input point cloud.

The proposed *LidPose* method is an end-to-end solution which solves the human detection and pose estimation task using only NRCS lidar measurements, in a surveillance scenario, where the sensor is mounted in a fixed position. The *LidPose* method’s workflow is shown in Figure 3.

First, the moving objects are separated from the static scene regions in the NRCS lidar measurement sequence by applying a foreground–background segmentation technique that is based on the *mixture-of-Gaussians (MoGs)* approach adopted in the range image domain, as described in [39]. A local background (Bg) model is maintained for each pixel of the range image, following the MoGs approach [42] applied for the range values. Due to the sparsity of the captured point clouds, within a given time frame, only the MoGs background model components of range image pixels corresponding to the actual measurement points are updated. The incoming measurement points are then classified as either foreground or background by matching the measured range values to the local MoGs distributions.

Second, the foreground point regions are segmented to separate individual moving objects, and the footprint positions of the detected pedestrian candidates are estimated. Here, a 2D lattice is fitted to the ground plane, and the foreground regions are projected to the ground. At each cell in the ground lattice, the number of the projected foreground points is counted; this is then used to extract each foot position, as described in [43]. The result of this step is a set of bounding boxes for the detected people, which can be represented both in the 3D space and in the 2D range image domain. As shown in [43], due to the exploitation of direct range measurements, the separation of partially occluded pedestrians is highly accurate; however, in a large crowd the efficiency of the approach can deteriorate.

In the next step, the NRCS lidar point cloud and the range image are cropped with the determined bounding boxes. The cropped regions correspond to lidar measurement segments containing points either from a person or from the ground under their feet.

To jointly represent the different available measurement modalities, we propose a new 2D data structure that can be derived from the raw lidar measurements straightforwardly and can be efficiently used to train and test our proposed LidPose model. More specifically, we construct from the input point cloud a five-channel image over the lidar sensor’s 2D range image lattice, where two channels directly contain the depth and intensity values of the lidar measurements, while the remaining three layers represent the X, Y, Z coordinates of the associated lidar points in the 3D world coordinate system.

Note that in our model, the pose estimator part of the method is independent of the sensor placement. While in this paper we demonstrate the application purely in a static lidar sensor setup, we should mention that with an appropriate segmentation method for a given scene, the *LidPose* pose estimation step could also be adapted to various—even moving—sensor configurations.

**Figure 4:**
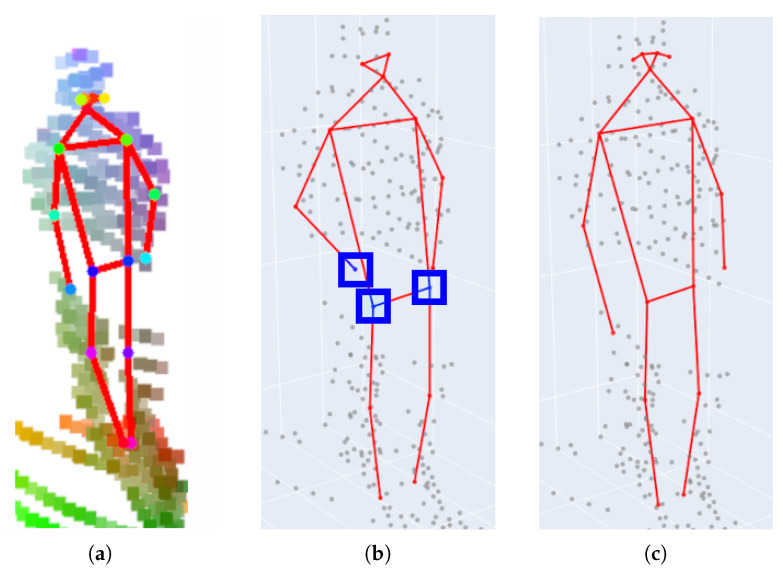
Predicted human poses of the *LidPose* variants, overlaid on the input data. (**a**) LidPose–2D: 2D predicted skeleton (red) over the 2D lidar point cloud representation (colored based on 3D coordinate value). (**b**) LidPose–2D+: 2D predicted skeleton (red) is extended to the 3D space using the lidar points (gray) where they are available. Points where lidar measurements are not available are highlighted in blue. (**c**) LidPose–3D: 3D predicted skeleton (red) over the lidar point cloud (gray).

To comprehensively explore and analyze the potential of using NRCS lidar data for the human pose estimation task, we introduce and evaluate three alternative model variants:

- *LidPose–2D* predicts the human poses in the 2D domain, i.e., it detects the projections of the joints (i.e., skeleton keypoints) onto the pixel lattice of the range images, as shown in Figure 4a. While this approach can lead to robust 2D pose detection, it does not predict the depth information of the joint positions.
- *LidPose–2D+* extends the result of the LidPose–2D prediction to 3D for those joints, where valid values exist in the range image representation of the lidar point cloud, as shown in Figure 4b. This serves as the baseline of the 3D prediction, with a limitation that due to the sparsity of the lidar range measurements, some joints will not be associated with valid depth values (marked by blue boxes in Figure 4b).
- *LidPose–3D* is the extended version of LidPose–2D+, where depth values are estimated for all joints based on a training step. This approach predicts the 3D human poses in the world coordinate system from the sparse input lidar point cloud, as shown in Figure 4c.

The ViTPose [28] network structure was used as a starting point in the research and development of the proposed LidPose methods’ pose estimation networks. Our main contributions to the proposed LidPose method:

- A new patch-embedding implementation was applied to the network backbone to handle efficiently and dynamically the different input channel counts.
- The number of transformer blocks used in the LidPose backbone was increased to enhance the network’s generalization capabilities by having more parameters.
- The output of the LidPose–3D configuration was modified as well by extending the predictions’ dimensions to be able to predict the joint depths alongside the 2D predictions.

As Figure 3 demonstrates, the *LidPose* network structure can deal with different input and output configurations depending on the considered channels of the above-defined five-layer image structure. The optimal channel configuration is a hyperparameter of the method that can be selected upon experimental evaluation, as described in detail in Section 4. In our studies, we tested the LidPose networks with the following five input data configurations:

- Depth only (D);
- 3D coordinates only (XYZ);
- 3D + depth (XYZ+D);
- 3D + intensity (XYZ+I);
- 3D + depth + intensity (XYZ+D+I).

For the training and testing of the proposed method, a new dataset was introduced, comprising an NRCS lidar point cloud segment and the co-registered human pose ground truth (GT) information for each sample object. The dataset is described in detail in Section 3. The three model variants introduced above are detailed in the following subsections.

#### 2.2.1. *LidPose–2D*

For pose estimation in the 2D domain, the *LidPose–2D* network was created based on the ViTPose [28] architecture. The patch-embedding module of the ViTPose backbone was changed to handle custom input dimensions for the different channel configurations (XYZ, D, I, and their combinations).

This new network architecture was trained end-to-end from an untrained, empty network. Five different networks were trained for the input combinations listed above. For these methods of predicting 2D joint positions, the training losses were calculated in the joint heatmap domain. An example of the *LidPose–2D* prediction can be seen in Figure 4a.

#### 2.2.2. *LidPose–2D+*

In this model variant, called *LidPose–2D+*, the 2D predictions created by *LidPose–2D* configuration are straightforwardly extended to the 3D space.

Each predicted 2D joint is checked, and if a valid depth measurement exists around the joint’s pixel location in the lidar range image, the 3D position of a given joint is calculated from its 2D pixel position and the directly measured depth value. This transfer from the 2D space to the 3D space implies a simple baseline method for 3D pose prediction models. However, the *LidPose–2D+* approach has a serious limitation originating from the inherent sparseness of the NRCS lidar point cloud. Two-dimensional joints whose positions are located in regions with missing depth measurements in the 2D range image cannot be extended to 3D. An example of a *LidPose–2D+* prediction is shown in Figure 4b, highlighting three joints that cannot be assigned to range measurements.

#### 2.2.3. *LidPose–3D*

The limitations of *LidPose–2D+* can be eliminated by a new network called *LidPose–3D* that aims to predict the depth of each detected joint, separately from its pixel position in the range image lattice. Similarly to the *LidPose–2D* variants described above, this network structure can handle inputs with different configurations of the XYZ, D, and I channels.

The *LidPose–3D* network’s output is constructed as an extension of ViTPose [28] to predict depth values for the joints alongside their 2D coordinates. The normalized depth predictions are performed on a single-channel 2D depth image in the same down-scaled image space (64×48) where the joint heatmaps are predicted. An example of a *LidPose–3D* prediction can be seen in Figure 4c.

### 2.3. *LidPose* Training

The training input data are a 2D array with a given number of channels—depending on the training configuration (combinations of XYZ, D, I). For the different channel configurations, different patch-embedding modules were defined to adopt the variable numbers of parameters in the input, as shown in Figure 3. For training and evaluation of the network, we also need the ground truth pose data, which we assume is available at this point. (Details of ground truth generation will be presented in Section 3).

Regarding the loss function of the *LidPose–2D* network, we followed the ViTPose [28] approach by using *mean squared error (MSE)* among the predicted and the ground truth heatmaps:

(1) $$L_{\mathrm{LidPose}--2D}:=L_{\mathrm{joint}2D}=MSE\left({\mathrm{HM}}_{\mathrm{pred}},{\mathrm{HM}}_{\mathrm{GT}}\right),$$

where ${\mathrm{HM}}_{\mathrm{pred}}$ and ${\mathrm{HM}}_{\mathrm{GT}}$ are the predicted joint heatmap and the ground truth joint heatmap, respectively.

For the *LidPose–3D* network, the training loss is composed of two components: one responsible for the joints’ 2D prediction accuracy ($L_{\mathrm{joint}2D}$), the other reflecting the depth estimation accuracy ($L_{\mathrm{depth}}$). The total training loss is a weighted sum of the position and depth losses:

(2) $$L_{\mathrm{LidPose}-3D}=W_{\mathrm{joint}2D}\cdot L_{\mathrm{joint}2D}+W_{\mathrm{depth}}\cdot L_{\mathrm{depth}}$$

For calculating the 2D joint position loss term $L_{\mathrm{joint}2D}$, Equation (1) was used again. Regarding the depth loss $L_{\mathrm{depth}}$, we tested three different formulas: *L1 loss*, *L2 loss*, and the *structural similarity index measure (SSIM)* [44]. Based on our evaluations and considering training runtime, the *SSIM* was selected for the depth loss measure in the proposed *LidPose–3D* network. Following a grid search optimization, the weighting coefficients in the loss function were set as $W_{\mathrm{joint}2D}=10$ and $W_{\mathrm{depth}}=1$.

## 3. Dataset for Lidar-Only 3D Human Pose Estimation

For the development and evaluation of the proposed *LidPose* method, we created a new dataset, since we have not found any public benchmark sets containing NRCS lidar measurements with human pose ground truth (GT).

GT annotation proved to be a challenging process since the visual interpretation of sparse 3D lidar point clouds is difficult for human observers, and the inhomogeneous NRCS pattern makes this task even harder. For facilitating ground truth generation and the analysis of the results, in our experimental configuration, a camera was mounted near the NRCS lidar sensor to record optical images in addition to the point clouds. The camera images were only used for creating the ground truth information for human pose estimation, and for helping the visual evaluation of the results of *LidPose*. During annotation, the operator used the camera images to mark, validate, and verify the skeleton joint positions.

During the dataset collection, the NRCS lidar (Livox Avia [10]) and the RGB camera were mounted together on a standing platform, and the measurement sequences were recorded in two outdoor and one indoor locations, where persons were walking in the sensors’ fields of view.

### 3.1. Spatio-Temporal Registration of Lidar and Camera Data

Since our experimental configuration uses both camera and lidar data for creating the ground truth human poses and validating the results, the spatial transformation parameters between the two sensors’ coordinate systems need to be determined by a calibration process.

The camera’s extrinsic and intrinsic parameters were calibrated using OpenCV [45,46] libraries and a Livox-specific, targetless calibration method [47]. The camera images were undistorted using the calibration’s distortion coefficients to remove the lens distortion and to provide rectified images for the dataset. Thereafter, the camera images and the lidar range images were transformed into a common coordinate system.

To establish the spatial correspondence between the camera and lidar sensors, the requirement of time synchronization of the data recordings arose. The camera and the lidar data were properly timestamped following the synchronization process described in the IEEE 1588 standard [48], using the *Precision Time Protocol daemon (PTPd)* [49], running on the data collector computer.

This enabled time-synchronous processing of the camera and the lidar sensor data with a precision of 1 ms. The camera and the lidar data were recorded with different, sensor-specific data acquisition rates, at 30 Hz on the camera and at 10 Hz in the case of the lidar. The corresponding image-point cloud pairs were created by selecting the camera image with the smallest time difference for each recorded lidar point cloud. In other words, the data collection was adjusted to the lidar’s slower frame rate.

### 3.2. Human Pose Ground Truth

Although the proposed *LidPose* method performs human pose estimation from solely NRCS lidar point clouds, in the ground truth generation phase we also took advantage of the co-registered camera images that were recorded in parallel with the lidar measurements.

#### 3.2.1. 2D Human Pose Ground Truth

The ground truth (GT) generation was implemented in a semi-automatic way, exploiting established camera-based person detection and pose-fitting techniques. In the first step, in each data sample, YOLOv8 [50] was run to detect the persons in the camera images. The detected persons’ bounding boxes with sizes smaller than ViTPose’s native input resolution (192×256) were discarded. The bounding box of a detected person was used to crop the person’s region both on the camera image and in the lidar data in the 2D range image domain.

In the second step, the initial pose estimation was created on the cropped camera images by the state-of-the-art 2D human pose estimator ViTPose [28] network with its *huge* configuration. This network configuration, where the network backbone has 32 transformer blocks, was selected based on its superior results in comparison to the smaller network variants. The trained model *ViTPose-huge* was obtained from the ViTPose [28] implementation from the repository at [51].

In the third step, the camera images were used to manually check, validate, filter, and fine-tune each 2D human pose, resulting in the 2D ground truth of human poses.

Since the lidar range images and the camera images were co-registered (both in time and space), the filtered camera-based pose models could be directly used as ground truth of the 2D human poses in the lidar’s range image domain. The skeleton parameters in the 2D ground truth were stored in the COCO-Pose [41] data format, which represents a given human pose with 17 keypoints, facilitating detailed pose estimation (see Figure 4).

#### 3.2.2. 3D Human Pose Ground Truth

The 3D human pose ground truth was created by the extension of the 2D human skeleton dataset, so that we attempted to assign to each joint a depth value based on the depth measurements of the lidar sensor around the joint’s 2D position. The challenge of this 2D-to-3D point assignment task arises from the sparseness of the measured NRCS lidar range image, which implies that some of the 2D joints cannot be assigned to genuine lidar depth measurements on the considered lidar frames. In these cases, we applied spatio-temporal interpolation, i.e., we interpolated the depth values of joints without direct range measurements from the depth values of other nearby joints, and from nearby frames.

### 3.3. Transforming the Point Cloud to the Five-Channel Range Image Representation

As described in Section 2, the LidPose method requires that the 3D lidar point cloud is transformed to a spherical polar coordinate system, using a 2D pixel lattice generated by quantizing the horizontal and vertical FoVs. The 3D world coordinates of the lidar points are stored in the 2D range image domain in different image channels.

As mentioned in Section 2.2, five different 2D data layers are created for each lidar point cloud. The first layer is the depth map, where values are the distances of the lidar points from the camera plane. The second layer is the intensity map, where the values are the reflection intensity of the lidar points. The remaining three layers store the coordinates of the lidar points in the 3D space ${\left(XYZ\right)}_{3D}$ at the calculated (u,v) range image locations.

### 3.4. Dataset Parameters

Independent recordings were made for the training, test, and validation datasets, where several moving pedestrians were observable in the sensors’ fields of view. One to three persons were walking at the same time following arbitrary directions in the observed field; meanwhile, they occasionally stopped during the movement, and some of them did gymnastic exercise-like activities. In parallel with the data capturing, the MoGs-based foreground–background segmentation method [39] was run on the lidar data, and the binary classification of the 3D points was stored for each frame alongside the camera and lidar measurements.

In total, our created new dataset contains 9500 skeletons and 161,000 joints. The dataset was split into the independent training, validation, and test sets, having 5500, 490, and 3400 skeletons, respectively, as shown in Table 1.

The training set consists of two sequences, both containing three individuals moving in a narrow courtyard. The validation set comprises two sequences which are recorded in a wide courtyard containing two individuals. The test set consists of three further sequences: The first one is recorded indoors, in a large room with a single observed individual. The second test sequence is captured on a wide courtyard with two subjects, and the third one is recorded on the same location with a single individual.

**Figure 5:**
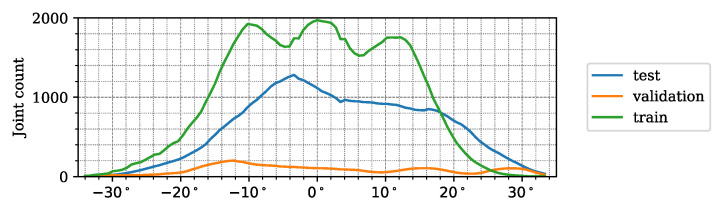
Distribution of the joints recorded in the *LidPose dataset*, based on the local emergence angle of the lidar sensor.

**Figure 6:**
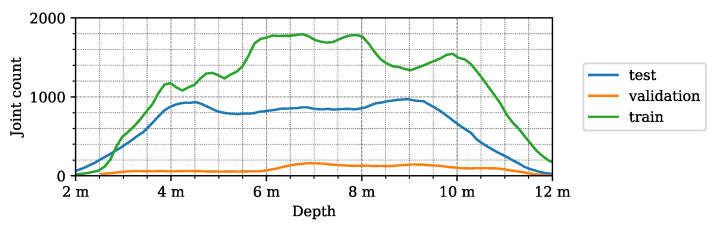
Distribution of the joints in the *LidPose dataset*, based on the depth coordinate (*X*) of the 3D joints.

**Figure 7:**
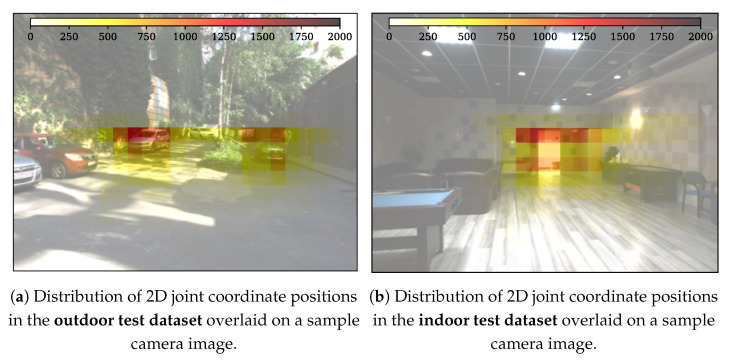
Distribution of 2D joint coordinate positions in the test dataset overlaid on a sample camera image.

**Figure 8:**
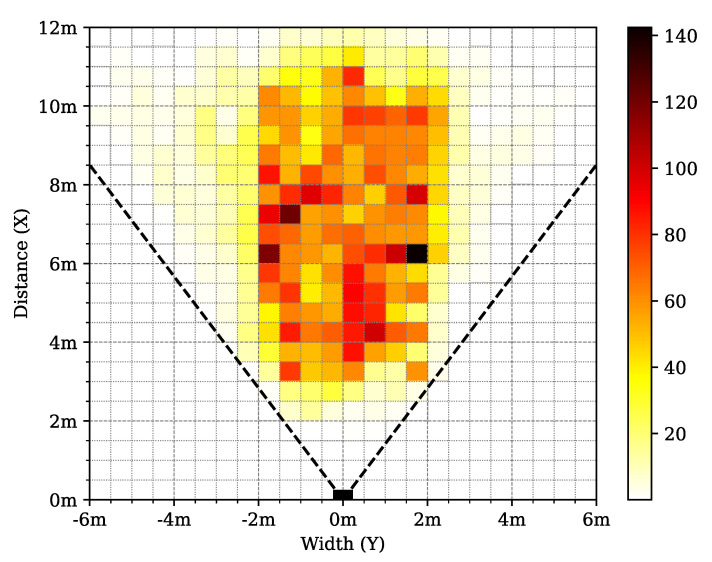
Distribution of joint positions in the *LidPose dataset*, displayed on the ground plane ${\left(X,Y\right)}_{3D}$ from a bird’s-eye view.

To support the deeper analysis and understanding of the structure and properties of our new dataset, we created the following graphical demonstrations. Figure 5 shows the number of joints in a given direction in the lidar FoV for the different datasets. It can be seen that the majority of the joint positions were recorded in the central 40° wide region of the lidar FoV. Figure 6 demonstrates the number of joints at a given depth $X_{3D}$ from the lidar sensor. Figure 7 presents the number of human poses displayed on the ground ${\left(XY\right)}_{3D}$ plane from a bird’s eye view. It demonstrates that as the observed people were crossing the sensor FoV, the central regions registered more skeletons than the regions near the FoV edge. Figure 8 shows the number of joints in the 2D camera image plane (u,v) in the pixel regions overlaid on a sample camera image. As the majority of the joints are recorded from the human torso, the regions more than 1 m above the ground registered more keypoints than the lower ankle and knee regions.

## 4. Experiments and Results

The proposed *LidPose* networks were trained to estimate human poses both in 2D and 3D. For *LidPose–2D*, five model variants were trained with different patch-embedding blocks on the corresponding input data configurations (D, XYZ, XYZ+D, XYZ+I, XYZ+D+I), as listed in Table 2 and Table 3.

Regarding *LidPose–3D*, we trained 12 model variants. On one hand, for each input configuration (XYZ, XYZ+D, XYZ+I, XYZ+D+I) the network was trained with different patch-embedding blocks. On the other hand, each configuration was trained with three different depth prediction losses: *L1, L2, and SSIM*. The trained models with their input and training loss are listed in Table 4.

**Table 2:** LidPose-2D network results on different input types with position loss. The meaning of the *input* values: D: lidar distance; XYZ: point 3D coordinates; I: lidar intensity; percentage of correct keypoints (*PCK*), calculated with the error being at most 10 pixels. The *AUC-PCK* was calculated on the [0, 30] pixel interval, as shown in Figure 9.

Model	Input	ADE ↓	PCK ↑	AUC-PCK ↑	LAE ↓	LLE ↓
2D–1	D	18.0726	0.4316	0.5360	13.7856	9.7695
2D–2	XYZ	14.4013	0.4960	0.5952	12.6956	9.5330
2D–3	XYZ+D	14.6881	0.4966	0.5926	12.7078	**9.4509**
**2D–4**	XYZ+I	**13.2473**	**0.5278**	**0.6166**	**12.5251**	10.6579
2D–5	XYZ+D+I	13.8399	0.5122	0.6049	12.6762	11.1547

**Table 3:** Mean per-joint position error (MPJPE) values of the LidPose–2D network for different joints.

Model	Head	Shoulders	Elbows	Wrists	Hips	Knees	Ankles	ADE ↓
2D–1	12.4838	17.5230	25.2190	27.6912	13.6437	16.6865	21.6442	18.0726
2D–2	11.3505	12.8925	17.3831	19.7888	10.9468	**13.7076**	**19.3161**	14.4013
2D–3	11.2303	13.1998	18.1070	20.5023	**11.2541**	14.0968	19.6134	14.6881
**2D–4**	**10.0393**	**11.1264**	**14.5071**	**17.0304**	11.5661	13.9160	19.3576	**13.2473**
2D–5	10.1436	11.6997	15.8984	18.6925	12.7537	14.1663	19.0698	13.8399

**Table 4:** Results of the *LidPose–3D* and *LidPose–2D+* networks with different input types and depth losses, evaluated in 3D space with 3D metrics. The meaning of the *input* values: D: lidar distance; XYZ: point 3D coordinates; I: lidar intensity; *Depth L*. refers to the criterion used to calculate the depth loss during learning. *2D+* models do not have this parameter. Percentage of correct keypoints (*PCK*) was calculated with the error being at most 0.2 m. The *AUC-PCK* was calculated on the [0, 0.5] meter interval.

Model	Input	Depth L.	ADE ↓	PCK ↑	AUC-PCK ↑	LAE ↓	LLE ↓
3D–01	XYZ	L1	0.3372	0.5222	0.5364	22.5130	0.9247
3D–02	XYZ	L2	0.1848	0.6904	0.6424	21.9994	0.0966
3D–03	XYZ	SSIM	0.1683	0.7322	0.6749	20.7884	**0.0903**
3D–04	XYZ+D	L1	0.2679	0.4599	0.5040	24.8060	0.1868
3D–05	XYZ+D	L2	0.1873	0.6784	0.6374	22.3703	0.0964
3D–06	XYZ+D	SSIM	0.1676	0.7374	0.6768	**20.6084**	0.0908
3D–07	XYZ+I	L1	0.2576	0.4822	0.5176	25.0920	0.1769
3D–08	XYZ+I	L2	0.1762	0.7147	0.6593	21.6107	0.1047
**3D–09**	XYZ+I	SSIM	**0.1583**	**0.7678**	**0.6942**	20.6737	0.0952
3D–10	XYZ+D+I	L1	0.2764	0.4164	0.4852	31.0437	0.2274
3D–11	XYZ+D+I	L2	0.1794	0.7014	0.6537	21.9404	0.1064
3D–12	XYZ+D+I	SSIM	0.1633	0.7466	0.6841	21.1505	0.0983
2D+–1	D	-	**2.4477**	0.4887	0.4427	**32.4529**	1.4299
2D+–2	XYZ	-	2.4758	**0.5242**	**0.4626**	32.5635	1.4419
2D+–3	XYZ+D	-	2.4910	0.5165	0.4583	33.3569	1.4723
2D+–4	XYZ+I	-	2.5901	0.5141	0.4534	34.0922	1.5538
2D+–5	XYZ+D+I	-	2.5671	0.5133	0.4541	33.5011	**1.3705**

**Figure 9:**
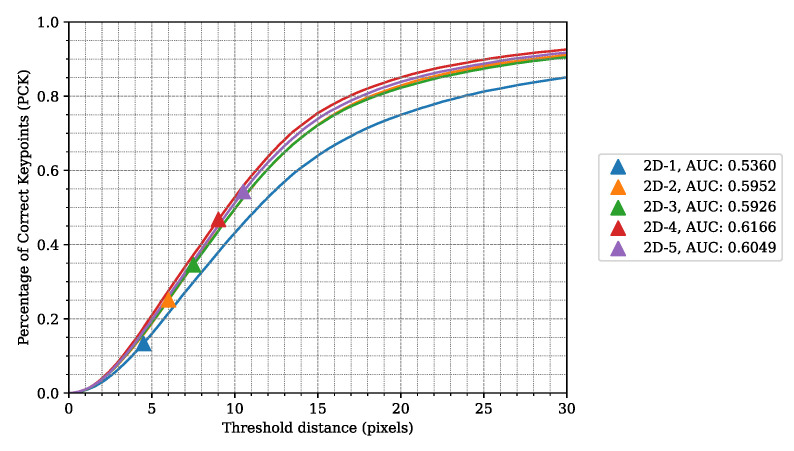
*LidPose–2D*: Percentage of correct keypoints for the different 2D networks with different joint-correspondence threshold acceptance values. Model *2D–4*, which is trained on 3D coordinates + lidar intensity, has the best PCK curve.

### 4.1. Metrics

The following metrics were calculated to compare the LidPose models. The visibility of a predicted joint *j* in skeleton *i* is represented by $v_{\left(i,j\right)}\in \left[0,1\right]$, indicating whether there is ground truth data for it. Thus, let *N* be the total number of visible joints in a given dataset:

$$N:=\sum_{i,j}v_{\left(i,j\right)}.$$

Additionally, let *Y* and $\hat{Y}$ be the ground truth and predicted coordinates of the keypoints, respectively.

Average distance error (ADE) measures the average Euclidean distance between the predicted pose and the ground truth pose across all skeleton joints, providing a measure of overall pose estimation accuracy. In the 2D case, normalization is applied based on the skeleton height to eliminate the varying skeleton sizes in the 2D image space. ADE, as defined in Equation (3). The lower the value, the better the performance.

(3) $$\mathrm{ADE}\left(Y,\hat{Y}\right)=\frac{1}{N}\sum_{i,j}v_{\left(i,j\right)}{∥Y_{\left(i,j\right)}-{\hat{Y}}_{\left(i,j\right)}∥}_{2}$$

Mean per-joint position error (MPJPE) [52] measures the position errors of different joint types. It is defined in Equation (4). MPJPE is similar to the ADE metric; however, it can highlight the performance differences between different body parts and regions.

(4) $$\mathrm{MPJPE}\left(Y,\hat{Y},J\right)=\frac{1}{\sum_{j}^{J}\sum_{i}v_{\left(i,j\right)}}\sum_{j}^{J}\sum_{i}v_{\left(i,j\right)}{∥Y_{\left(i,j\right)}-{\hat{Y}}_{\left(i,j\right)}∥}_{2},$$

where *J* is a subset of all joints.

Percentage of correct keypoints (PCK) [53] shows the percentage of joints in the estimated pose that fall within a certain threshold distance from their corresponding ground truth keypoints. In the 2D space, the distance threshold is set in pixels, while in the 3D space, it is set in meters. This measure, defined in Equation (5), assesses the accuracy of joint localization at different levels of precision; the higher the value, the better the prediction.

(5) $$\mathrm{PCK}\left(Y,\hat{Y},\alpha \right)=\frac{1}{N}\sum_{i,j}\delta_{\left(i,j\right)}\left(\alpha \right),$$

where α is the error threshold and δ is an indicator function.

(6) $$\delta_{\left(i,j\right)}\left(\alpha \right):=\left\{\begin{array}{cc} 1 & \mathrm{if}\;∥Y_{\left(i,j\right)}-{\hat{Y}}_{\left(i,j\right)}{∥}_{2}\leq \alpha \\ 0 & \mathrm{otherwise} \end{array}\right.$$

The PCK curve can be constructed by sweeping the distance threshold.

The *area under curve (AUC)* value of a PCK curve is a good generalizing metric for human pose estimation tasks [54]. The PCK evaluates the performance of an examined human pose estimation method based on a single threshold; PCK-AUC on the other hand uses a series of thresholds, providing a more comprehensive assessment of the method’s performance. This also reduces the sensitivity of the results to the choice of the parameter.

Limb angle error (LAE) calculates the mean angular difference between the orientations of corresponding limbs (arms, legs) in the predicted skeleton and the ground truth skeleton, as defined in Equation (7). It assesses the accuracy of orientation estimation both in the 2D and 3D spaces.

(7) $$\mathrm{LAE}\left(Y,\hat{Y},L\right)=\frac{1}{\sum_{i}v_{i}^{L}}\sum_{i}\left|\mathrm{angle}\left(Y_{i},L\right)-\mathrm{angle}\left({\hat{Y}}_{i},L\right)\right|,$$

where *L* is a subset of joints that has three elements that are connected by the skeleton edges, and $v_{i}^{L}\in$ [0, 1] indicates whether the whole limb is present in the prediction and ground truth for a given skeleton. *angle()* calculates the angle of the skeleton edges at the middle joint of the limb.

Limb length error (LLE) was calculated on skeleton limbs (arms, legs) to measure how the network predicts their total length, as defined in Equation (8). This measure does not penalize if the elbow or the knee is not predicted accurately until the total limb length is estimated correctly.

(8) $$\mathrm{LLE}\left(Y,\hat{Y},L\right)=\frac{1}{\sum_{i}v_{i}^{L}}\sum_{l}^{L}\sum_{i}\left|\right|Y_{\left(i,l\right)}\left|-\right|{\hat{Y}}_{\left(i,l\right)}\left|\right|,$$

where *L* and $v_{i}^{L}$ are the same as in Equation (7).

### 4.2. Experiment Parameters

**Figure 10:**
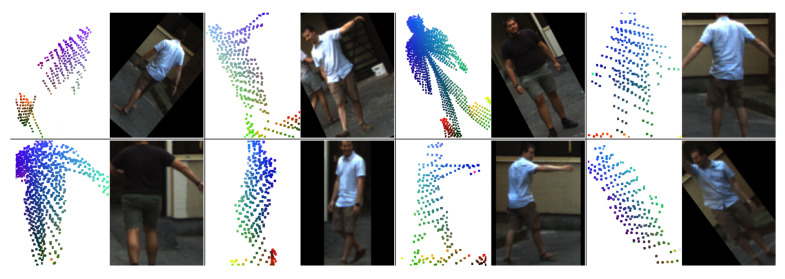
Example training batch of input data with the randomly applied augmentations (horizontal mirroring, scaling, rotation, half-body transform). The camera images are shown for visual reference only.

During the training of the *LidPose* models, data augmentation was applied both to the five-channel 2D input arrays and the ground truth skeletons. Vertical mirroring, scaling, and rotation transforms were added to each data sample randomly to enhance model robustness and estimation efficiency. To enhance the network’s robustness on partial skeletons, *half-body transform* was applied randomly during the training process, where either the upper body or the lower body of a skeleton was selected and cropped, as in [28]. Figure 10 shows a batch of input data with the randomly applied augmentations mentioned above.

During the training of *LidPose*, AdamW was used with a weight decay coefficient of λ=0.1 and $\beta_{1}=0.9$ and $\beta_{2}=0.999$. The maximum learning rate was set to $\gamma =5\cdot 10^{-4}$; this was reached after three batches with a ramp-up. Learning rate decay was used to decrease the learning rate exponentially by a factor of 0.1 between epochs 20 and 30, 30 and 35, and 35 and 100.

For both training and inference, two types of computers were used: a set of desktop computers having 12/16 CPU threads, 32 GB RAM and 11 GB vRAM in Nvidia GeForce 1080Ti GPU, and a cloud computer instance in HUN-REN Cloud [55] with 8 vCPU cores, 32 GB RAM, and 16 GB vRAM in an Nvidia Tesla V100 GPU cluster. The training was run with a batch size of 48, and one step took 5 s on both types of computers. The networks were trained for 100 epochs.

The proposed *LidPose* runs at 52 FPS on the prerecorded dataset in offline processing on singleton batches. In the end-to-end application of the proposed pipeline, the frame rate of the method is determined by the NRCS lidar’s sampling rate (10 FPS).

### 4.3. *LidPose–2D* Evaluation

The evaluation results based on the metrics described in Section 4.1 are shown in Table 2 and Table 3. The test results show that model *2D–4* outperforms the other model variants with almost all the metrics for the 2D human skeleton estimation task. This best model variant corresponds to the XYZ+I channel configuration, i.e., it uses the 3D point coordinate values and the lidar reflection intensity.

From Table 2 it can be seen that the depth-only method (*2D–1*) has weak performance, as the network does not have enough information to estimate the 2D skeleton positions accurately. If the input of the *LidPose–2D* network is the 3D point coordinate data in three input channels (*2D–2*), the ADE and the LAE decrease significantly. The combination of the two formers, i.e., the depth values and the 3D joint coordinates used as the input, model variant *2D–3*, achieves the lowest LLE. If the previous variant is extended by the lidar intensity (*2D–5*), the network does not outperform the *2D–4* network variant, as the former achieves 13.84 px ADE, while the latter scores 13.2 px ADE.

**Figure 11:**
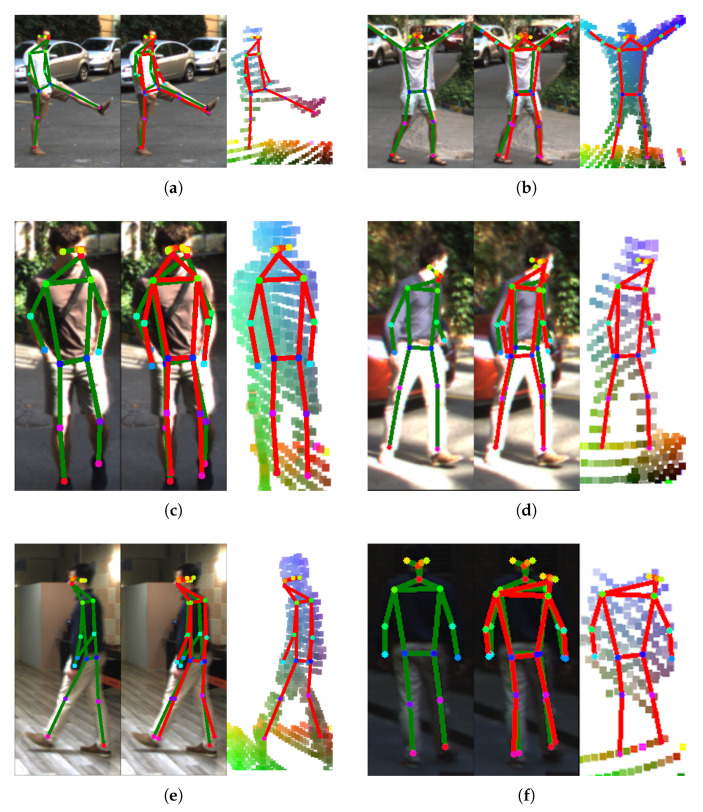
*LidPose–2D* prediction examples are shown in subfigures (**a**–**f**) for different samples from the dataset. The predictions are shown in red, overlaid on the input lidar point cloud (right). The corresponding camera frame, and the ground truth is shown in green (left). The prediction and the ground truth are shown together overlaid on the camera image (middle).

Table 3 lists the MPJPE values for the different LidPose–2D model variants. It can be seen that the torso joints (head, shoulders, hips) have lower MPJPE scores than the limb-related joints. This can be explained by the smaller size of those parts, and thus, there being fewer or no measurements in the sparse lidar point cloud at those locations. An example of this can be seen in the left leg of the person in Figure 11.

Figure 9 shows the PCK values of each 2D model for different threshold values. The AUCs of these PCK graphs were calculated (also shown in Table 2), where *model 2D–4* has the highest score.

**Figure 12:**
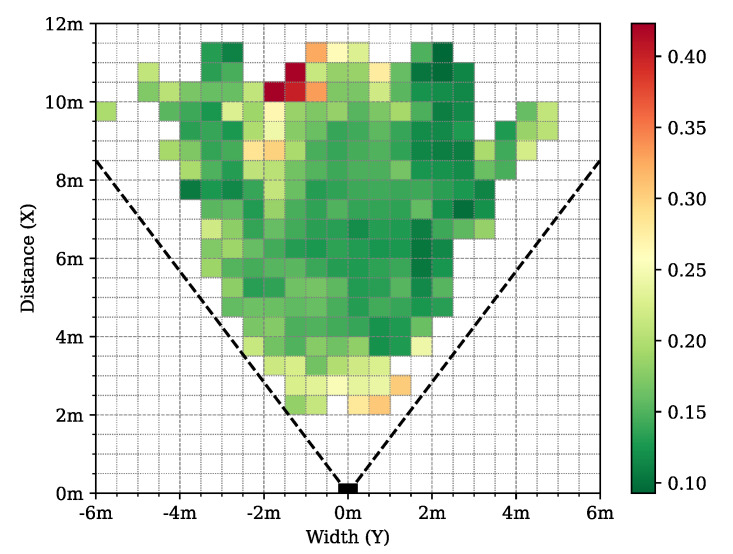
2D average distance error (ADE) of the selected *2D–4* model, overlaid on a sample camera image.

The ADE of the selected model was evaluated in different 2D image regions, as shown in Figure 12. From this figure it can be seen that as the 2D estimation positions in this 2D camera image space become closer to the edge of the lidar’s FoV, the ADE value increases above 50 pixels, meanwhile in the central regions the ADE score is below 20 pixels. This behavior is the consequence of the inhomogeneous nature of the NRCS lidar point cloud, where the point cloud sparseness increases with the distance from the sensor’s optical center.

Example estimations are shown in Figure 11, where the ground truth is shown in the camera image, and the lidar-based 2D skeleton prediction is displayed on the sparse point cloud. Figure 11b,c show skeletons where the human was at a distance of 5 m from the lidar, resulting in less sparse point clouds. On the contrary, Figure 11a,d,e show skeletons at 10 m distance, leading to far fewer lidar points in the frame. It can be observed that the skeleton estimation accuracy is high, as the predicted and the ground truth are very close. Figure 11f shows an example where the prediction makes a mistake on the person’s head as there are no recorded 3D points from that region in that given frame.

### 4.4. *LidPose–3D* Evaluation

**Figure 13:**
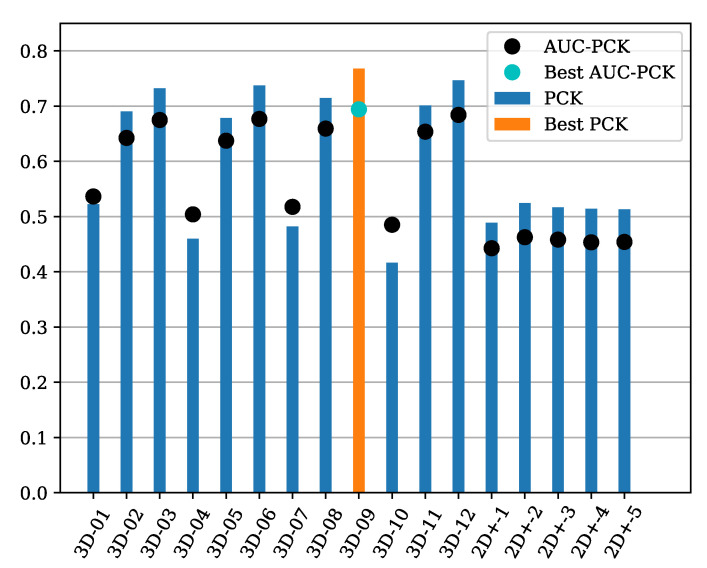
PCK and AUC-PCK values of the 3D predictions by LidPose–3D and LidPose–2D+ networks evaluated in 3D space with 3D metrics. The *AUC-PCK* was calculated on the [0, 0.5]-meter interval, as shown in Figure 14.

The *LidPose–3D* networks predict the 2D joint positions in the same manner as LidPose–2D, and the depth values for each joint. From the predicted 2D position and the depth values the 3D joint positions are calculated. The results are evaluated using various 3D metrics in the 3D space, as described in Section 4.1. The baseline of the 3D evaluation is the *LidPose–2D+*, described in Section 2.2.2. Table 4 and Table 5 and Figure 13 show the results for both the LidPose–3D and LidPose–2D+ models. As we can see, the 3D models are considerably better overall.

**Figure 14:**
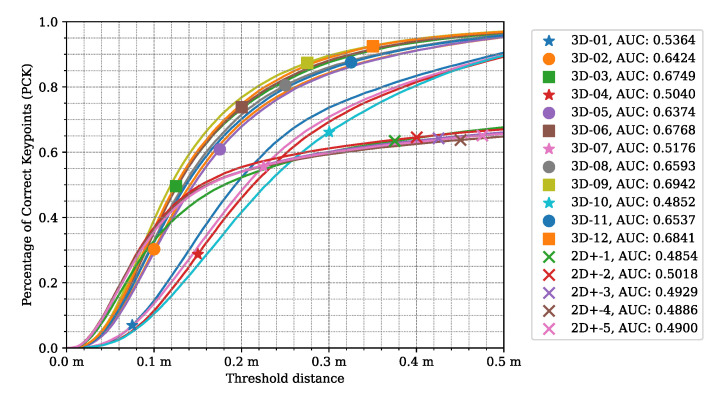
*LidPose–3D*: percentage of correct keypoints (PCK) in the 3D space for the different 3D (and 2D+) networks with different joint-correspondence threshold distance acceptance values. *Model 3D-9*, which has been trained on 3D coordinates + lidar intensity with SSIM-based depth loss, has the best PCK curve.

Upon assessing the PCK values of the 3D models in Figure 14, the models can be grouped based on their PCK curve shape.

Group one consists of models that did not learn the depth estimation properly during training. Namely, *3D–01, 3D–04, 3D–07*, and *3D–10* failed to learn depth estimation. Their common attribute is that they were using L1 loss to penalize the depth error during the learning process.

The second group contains the projected 2D models (LidPose–2D+), described in Section 2.2.2. These models all perform very similarly to each other while performing distinctly from the other two groups. These models serve as a baseline for the proposed method. Their performance is equal to or better than the 3D models in the 0–0.1 m interval, as they have significantly more correct predictions than at a larger distance. This is due to the assembly of the 3D predictions from existing 3D points at the predicted 2D joints’ positions. These characteristics highlight that while this approach works well with sparse but homogeneous lidar measurements, as shown in [14], it fails on point clouds recorded with NRCS lidar.

Lastly, the third group is the rest of the 3D models, which use L2 loss and SSIM as the depth criterion. As can be seen, these models correctly estimate the human poses, and the trend is similar to the 2D models in Figure 9. Notably, while the shape of these curves is similar, models with the SSIM-based depth loss outperform the models trained with L2 loss. *Model 3D–09* outperforms all other configurations.

**Figure 15:**
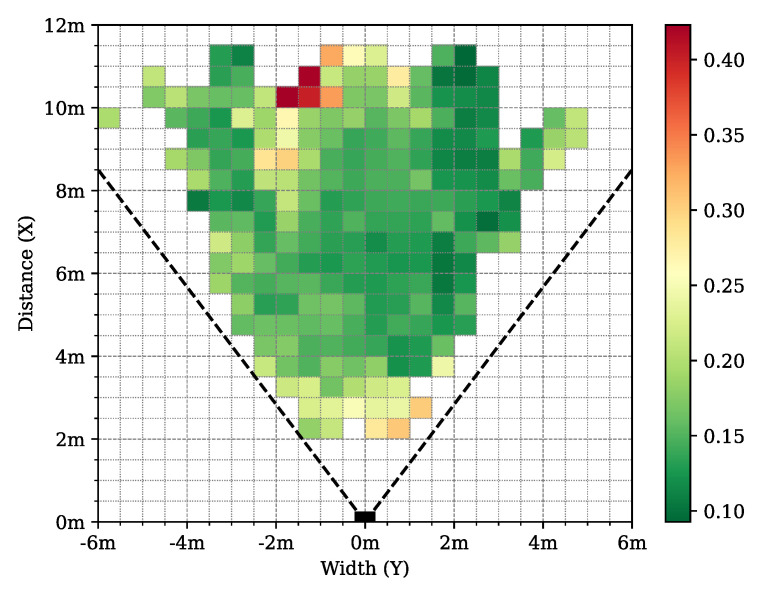
Distribution of average distance error (ADE) of the predicted joints in bird’s eye view using the selected *3D–09* model. Only cells with more than 24 annotated joints are shown.

The best 3D network, *3D–09* was evaluated with the ADE metrics on the ground plane on the test dataset to show the spatial dependency of the prediction performance at different regions. Although the maximum ADE error is 0.5 m, most of the cells of the ground grid have less than 0.3 m average error rates, as shown in Figure 15.

Table 5 shows the MPJPE results for the 3D methods. It can be seen that the projected 2D+ models (LidPose–2D+), described in Section 2.2.2, are outperformed by all the LidPose–3D networks.

The 2D models in Table 2 were projected to 3D prediction using the inhomogeneous sparse lidar data. This was achieved by using nearby 3D data where it was available for the back-projected 2D predictions. However, due to the characteristics of the NRCS lidar sensor, this approach has its limitations. Figure 13 and Figure 14 and Table 4 also show that *LidPose3D* outperforms the extended *2D+* networks.

**Figure 16:**
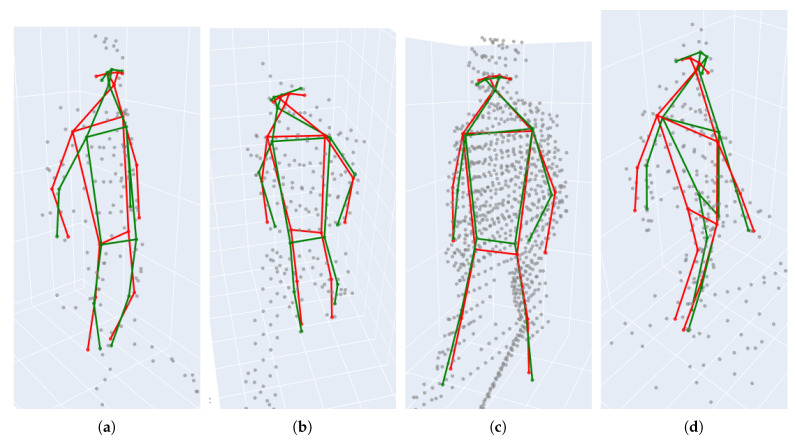
*LidPose3D* prediction examples are shown in subfigures (**a**–**d**) for different samples from the dataset, using the *3D–09* model. Red skeleton: 3D prediction. Green skeleton: ground truth. Gray points: NRCS lidar points.

In Figure 16, 3D human pose samples are shown from different viewing angles. By inspecting Figure 16a,b, it can be seen that there is a correlation between the density of the points and the accuracy of the network. This angle and distance dependency can also be observed in Figure 12 and Figure 15.

The experiments in this section have shown that the proposed *LidPose* methods are capable of the efficient and accurate estimation of the human poses. Our obtained results provide strong evidence that the NRCS lidar sensor is suitable for solving the lidar-only 2D and 3D human pose estimation tasks.

## 5. Conclusions and Future Work

In this paper, a method was introduced for real-time human pose estimation from inhomogeneous and sparse point clouds recorded by a non-repetitive circular scanning (NRCS) lidar sensor, called Livox Avia. To train and test the method, a novel camera and NRCS lidar-based dataset was created with ground truth pose models. The proposed *LidPose* method belongs to a vision-transformer-based neural network family, and we also demonstrated that it can be incorporated into an end-to-end workflow of person detection and human pose estimation for surveillance scenes.

The obtained results confirm that the proposed method is capable of detecting human skeletons in sparse and inhomogeneous NRCS lidar point clouds. Our approach gives accurate human pose estimation results in real time in the 3D world coordinate system of the scene, which can be used in higher-level scene analysis steps of surveillance systems. Thus, the paper also gives evidence that this NRCS lidar, which can be widely adopted in real-life scenarios due to its low price, can be used for solving complex human pose estimation tasks, while the process highly respects the observed people’s privacy as the people are barely recognizable by human observers from the recorded sparse point clouds.

As future work, we intend to transfer the proposed LidPose approach to a moving platform (robot, vehicle, etc.) by replacing the foreground detection-based preprocessing step with a geometric or deep-learning-based point cloud segmentation method to select and crop the input data for the pose estimator neural networks.

## Figures and Tables

**Table 1:** Overview of the distributions of the *LidPose* dataset over its train, validation, and test splits.

Dataset	Count		Location Mean, STD (*m*)		
	**Joint**	**Skeleton**	**X**	**Y**	**Z**
Train	94,248	5544	7.34 (±2.27)	0.13 (±1.29)	−0.54 (±0.54)
Validation	8364	492	7.59 (±2.26)	−0.05 (±2.22)	−0.50 (±0.52)
Test	59,228	3484	6.86 (±2.28)	−0.25 (±1.55)	−0.55 (±0.52)
Total	161,840	9520			
Average			7.26 (±2.27)	−0.06 (±1.69)	−0.53 (±0.53)

**Table 5:** Mean per-joint position error (MPJPE) results of the LidPose–3D networks for different joint types.

Model	Head	Shoulders	Elbows	Wrists	Hips	Knees	Ankles	ADE↓
3D–01	0.1693	0.2252	1.1299	0.3677	0.1741	0.2045	0.3413	0.3372
3D–02	0.1368	0.1537	0.2038	0.2252	0.1374	0.1768	0.3320	0.1848
3D–03	0.1375	0.1404	0.1694	0.1933	0.1368	0.1611	0.2860	0.1683
3D–04	0.1817	0.2512	0.3898	0.3914	0.2009	0.2334	0.3560	0.2679
3D–05	0.1410	0.1576	0.2063	0.2297	0.1386	0.1767	0.3305	0.1873
3D–06	0.1386	0.1391	0.1654	0.1898	0.1347	0.1591	0.2899	0.1676
3D–07	0.1698	0.2214	0.3676	0.4060	0.1999	0.2239	0.3466	0.2576
3D–08	0.1323	0.1442	0.1762	0.2040	0.1369	0.1705	0.3349	0.1762
**3D–09**	**0.1290**	**0.1272**	**0.1509**	**0.1734**	**0.1303**	**0.1585**	**0.2827**	**0.1583**
3D–10	0.1853	0.2282	0.3865	0.4642	0.2308	0.2387	0.3372	0.2764
3D–11	0.1332	0.1486	0.1819	0.2085	0.1430	0.1771	0.3330	0.1794
3D–12	0.1309	0.1347	0.1568	0.1813	0.1399	0.1625	0.2855	0.1633
2D+–1	**2.4256**	1.6951	2.2149	3.2206	**1.9731**	2.4557	3.1819	**2.4477**
2D+–2	2.5820	**1.6506**	**2.1371**	3.1362	2.0028	2.4956	3.1668	2.4758
2D+–3	2.5793	1.6879	2.2053	3.1573	1.9953	2.4664	3.2130	2.4910
2D+–4	2.8987	1.7478	2.2795	3.1611	2.0405	2.4757	**3.0646**	2.5901
2D+–5	2.9546	1.7358	2.1397	**3.0070**	2.0248	**2.4215**	3.1053	2.5671

## Data Availability

The data presented in this study are available on request from the corresponding author due to privacy restrictions.

## Abbreviations

The following abbreviations are used in this manuscript:

ADE	Average distance error
AUC	Area under curve
Bg	Background
COCO	Microsoft COCO: Common Objects in Context dataset
FFN	Feed-forward network
FoV	Field of view
FPS	Frames per second
GT	Ground truth
IMU	Inertial measurement unit
HUN-REN	Hungarian Research Network (https://hun-ren.hu/en)
LAE	Limb angle error
LLE	Limb length error
MHSA	Multi-head self-attention
MRF	Markov random fields
MoGs	Mixture of Gaussians
MPJPE	Mean per-joint position error
MSE	Mean squared error
NRCS	Non-repetitive circular scanning
PCK	Percentage of correct keypoints
PTPd	Precision Time Protocol daemon
ReLU	Rectified linear unit
RoI	Region of interest
RMB	Rotating multi-beam
SLAM	Simultaneous localization and mapping
SSIM	Structural similarity index measure
